# The Impact of Physical Activity Levels and Functional Fitness Status on the Quality of Life Perceived by Older Adults Living in Rural and Urban Areas: The Portuguese Inland Case

**DOI:** 10.3390/healthcare10071266

**Published:** 2022-07-07

**Authors:** Guilherme Eustáquio Furtado, Cláudia Vaz, Antonio Bovolini, Ermelinda Marques, Nuno Serra, Ana Raquel Costa-Brito, Carolina Vila-Chã

**Affiliations:** 1 Research Unit for Inland Development (UDI), Polytechnic of Guarda, 6300-559 Guarda, Portugal; guilhermefurtado@ipg.pt (G.E.F.); claudiasvaz92@gmail.com (C.V.); bovolini@sal.ipg.pt (A.B.); emarques@ipg.pt (E.M.); nserra@ipg.pt (N.S.); raquelbrito@ipg.pt (A.R.C.-B.); 2 Center for Health Technology and Services Research (CINTESIS), 4200-450 Porto, Portugal; 3 Research Center in Sports Sciences Health and Human Development (CIDESD), 5001-801 Vila Real, Portugal

**Keywords:** health-related quality of life, handgrip strength, short physical performance battery, socio-environmental factors

## Abstract

Health-related quality of life (HRQoL) is influenced by several factors, such as living place, physical activity (PA), and functional fitness levels. Evidence shows that functional fitness and PA levels are strongly associated with positive HRQoL, especially in the older population. However, the impact of the living place has not been investigated as an influencing variable in this context. Therefore, this study aimed to investigate the relationship between the HRQoL, PA, and functional fitness of older adults living in rural and urban areas of Portugal. A cross-sectional study was performed with community-dwelling adults aged 65 years and over (*n* = 261) living in the city of Guarda. The participants were assessed for sociodemographic, anthropometric, clinical health, HRQoL, PA levels, and functional fitness status. The results showed that rural residents presented higher scores of HRQoL and functional fitness than older individuals living in urban areas. Regression models showed that functional fitness variables influence the HRQoL overall score and mental and physical subcomponents of HRQoL, regardless of the living place. In contrast, PA levels only influenced the HRQoL score in rural residents. The findings suggest that intervention programs to improve the physical health, quality of life, and well-being of the older population need to consider the country’s different geographical areas.

## 1. Introduction

In the last decades, a steep increase in life expectancy of the older population has been observed, which does not always reflect an increment in the years lived with quality of life after 65 years old [1]. In this sense, assessing the health-related quality of life (HRQoL) is considered very useful to describe the life experiences of older adults as it relates to their physical and mental health status [2]. For this reason, health professionals have increasingly used HRQoL measurements to identify older adults’ health risks and define effective interventions [3]. Previous studies have shown that older adults with poor HRQoL are at greater risk of developing more severe chronic diseases and early mortality [4,5]. Furthermore, physical and cognitive frailty syndromes were also negatively associated with HRQoL [6,7].

The maintenance or improvement of the HRQoL and functional abilities are influenced by several factors, including physical activity (PA) levels [8] and physical-functional fitness status [9,10]. Physical-functional fitness refers to the ability of the body to adapt to the external environment and cope with daily life activities, and PA is understood as any bodily movement produced by skeletal muscles that require energy expenditure [11]. The term “physical inactivity” represents the non-achievement of PA guidelines, defined as less than 150 min of moderate-intensity activity per week or equivalent [12]. Both physical fitness and PA levels are typically assessed using validated methodologies and instruments [11]. For example, accelerometers and questionnaires are the most common instruments to assess PA levels, whereas physical-functional fitness is typically measured with different instruments and methodologies, considering their sub-dimensions, such as body composition, cardiovascular endurance, muscular strength, and endurance [8,11].

Physical-functional fitness improvements associated with regular practice of PA can increase older adults’ HRQoL [13,14,15,16] and life satisfaction [17]. Several studies demonstrated that high handgrip strength levels are negatively associated with disability, sarcopenia, and morbidity [18,19,20], influencing the perceived HRQoL of older adults [16]. Moreover, muscle strength and endurance losses in the lower limbs have been associated with imbalance [21], and increased risk and fear of falling [22].

Despite the positive effects of PA and physical fitness status on health and HRQoL [9,13], poor levels of PA [23] and physical fitness [24] are highly prevalent among older adults [21,22]. Due to the aging process, a reduction in PA levels has been associated with earlier deterioration in functional-physical fitness capacities [8] and a high incidence of non-communicable diseases over time [25,26]. Several factors directly or indirectly related to PA and physical fitness have been previously identified [11,27,28]. Among them, advanced age, physical limitations, poor cognitive status, low social support, and depression are major sociodemographic risk factors associated with physical inactivity behavior [29]. In Portugal, a study with a representative sample of adults (18 to 64 years) revealed that being male, young, and highly educated were factors associated with high levels of physical activity [30]. These sociodemographic factors also influence HRQoL in older individuals [31,32]. Furthermore, clinical health status seems to be a key determinant of HRQoL [31], and influences both PA and physical fitness status [33].

Besides the well-known influence of these variables on HRQoL and physical health status in older people, there is some evidence that older rural residents experience a health disadvantage compared to urban residents [34]. Nonetheless, there is no consistent evidence that older people living in rural areas have low scores of physical fitness, PA, and HRQoL compared to those living in urban areas [35,36,37,38]. For instance, in studies performed in China (*n* = 1029) and Brazil (*n* = 2292), the urban older residents showed greater HRQoL than their rural counterparts [39,40]. On the other hand, a large community survey study conducted in Italy (*n* = 685) reported that older rural residents perceived HRQoL more positively than urban residents [41]. Studies carried out in Portugal (*n* = 483), and Serbia (*n* = 100) revealed that older adults living in both urban and rural areas perceived HRQoL similarly [42,43].

Conflicting results regarding the impact of living in rural versus urban areas on HRQoL have been previously reported [40,41,43]. In addition, whether PA levels or functional-physical fitness status better predicts HRQoL levels remains to be established. A study conducted in Turkey (*n* = 174) showed that the living environment did not influence the physical function status and HRQoL of older people [44]. In this study, no associations between the HRQoL and physical function variables were presented [44]. A study conducted in Brazil also showed no differences between rural and urban areas regarding HRQoL and PA [45]. On the other hand, in another study conducted in Brazil, a positive correlation between PA levels and the HRQoL was found in older adults living in rural areas [45]. The authors also observed that older persons from the rural area, who were regularly active, had higher HRQoL scores than urban individuals. Conversely, in a Japanese study, urban participants had better HRQoL scores than rural ones, and PA was associated with their perception of HRQoL [46].

The contradictory results may be explained by distinct environmental features related to rural and urban settings, but also to specific characteristics of the population (i.e., sociodemographic and clinical status). In this sense, the implementation of global guidelines implies acting locally by understanding the specific contexts. Thus, this study aimed to investigate differences in the perceived HRQoL of older adults living in rural and urban areas, and its association with PA levels and functional-physical fitness status. Based on the assumptions presented above, we hypothesised: (i) the HRQoL perceived by older adults differs between rural and urban contexts; (ii) both PA levels and functional-physical fitness status present significant positive correlations with HRQoL; (iii) sociodemographic, anthropometric, and clinical health status affects the strength of correlations.

## 2. Materials and Methods

### 2.1. Study Design

This cross-sectional study was part of an epidemiological research project that assessed PA levels, HRQoL, physical-functional fitness status, clinical health status, and barriers to PA in community-dwelling older adults (GMove+*,* Guarda in Motion to Promote Active Ageing).

### 2.2. Participants and Settings

Data collection was carried out between February 2017 and March 2018 in the municipality of Guarda, localised in the Inland region of Portugal. Approximately 400 community-dwelling older adults were contacted, and 317 of them accepted the invitation to participate in the first stage of the study. Participants were recruited through the dissemination made at public health centres in collaboration with local physicians and health professionals. Participants were included if they (i) were older than 65 years, (ii) did not have any chronic disease that impaired their mobility and daily life tasks, (iii) lived in their home, and (iv) accepted participating in the study.

Exclusion criteria of participants comprised: (i) having difficulty moving with full autonomy; (ii) having cognitive limitations that affected the comprehension and performance in the psychometric tests (questionnaires); and (iii) living in social and health care support centres. A total of 259 participants, residing in urban (*n* = 139, 54%) and rural (*n* = 120, 46%) areas, were included in the data analysis. Personal data were controlled anonymously, and ethical approval was granted by the Ethics Committee of the Local Health Unit of Guarda (no 11136) in compliance with the guidelines for research with human beings of the Helsinki Declaration [47]. Before data collection, all participants were informed about the purpose of the project, with informed consent signed.

### 2.3. Outcomes Measures

Data collection was performed by a trained research team. The HRQoL, PA levels, and functional-physical fitness status were treated as central outcomes. The sociodemographic, anthropometric, and clinical health status variables were treated as covariates (in the regression model), following the evidence of previous studies, which demonstrated the influence of these indicators on HRQoL [31,48,49].

### 2.4. Central Outcomes

#### 2.4.1. Health-Related Quality of Life Questionnaire

Health-related quality of life (HRQoL) was assessed using the Portuguese version of the SF-36 questionnaire [50]. The SF-36 consists of 36 items that were used to compute scores on eight dimensions of HRQoL (physical functioning, physical health, bodily pain, general health status, vitality, social functioning, emotional health status, and mental health), and measure changes in health status as a ninth dimension [51]. The scores range between 0 and 100, with a higher score indicating a better HRQoL [52,53]. Based on the score of eight dimension scores, the HRQoL overall score, and physical component (PCS) and mental component (MCS) summaries were generated, through the sum of the standardised scores of each dimension, according to the respective coefficients of the MCS and PCS subcomponents [53], previously described in the questionnaire validation study for the Portuguese population [54]. This scale presented high internal consistency and reliability in the validation process, with Cronbach alpha (α) ranging from 0.80 to 0.86 [50].

#### 2.4.2. Physical Activity Levels

A Portuguese version of the Yale Physical Activity Survey (YPAS-p) for older adults was used to assess PA levels [55]. Activity dimension indices were computed by multiplying the frequency score by the duration score for each of the five categories, posteriorly multiplied by a weighting factor [53]. The summary index corresponds to the sum of these five individual indices [56]. Good internal consistency (α ≥ 0.83) and temporal stability (ICC > 0.75) were reported in the Portuguese version of the YPAS-p transcultural validation study [57].

#### 2.4.3. Physical-Functional Fitness Status

The Short Physical Performance Battery (SPPB) and Handgrip Strength Test (HGT) were used to evaluate the physical-functional fitness status [58]. The SPPB test is composed of the following tests: (i) the standing balance test, which consists of maintaining (for 10 s) the following 3 different foot postures: side-by-side (position 1), semi-tandem (position 2), and tandem stance (position 3); (ii) the 4 metres test to assess gait speed, consisting of two attempts to walk; (iii) the time necessary by each participant to complete five full chair stands with the arms folded across the chest [59]. The SPPB score varies between 0 and 12 points (each task scoring from 0 to 4 points). A score of 0 represents an inability to carry out the test, and a score of 12 is the best test performance. The final sum assigns a categorical classification, indicating a very poor (0–3 points), a low (4–6 points), a moderate (7–9 points), or a good (10–12) capacity of participants performing the battery tests [58,59]. The HGT was measured with a portable hydraulic dynamometer (Jamar^®^, Lafayette, LA, USA). The participants were asked to grip the dynamometer as hard as possible for 5 s, with the arm extended at the side of the body, in a standing position, without pressing the instrument against the body or bending at the elbow. Three trials were performed with each hand, with a brief rest between the trials. The best performance was used for the analysis.

### 2.5. Covariates

#### 2.5.1. Sociodemographic Status

Sex was based on participant self-identification and coded as a binary variable: female and male. Age was treated as a continuous variable. Level of education was assessed as a categorical variable according to the Portuguese Educational System [60], and 3 subcategories were created: <4th Grade, = 4th Grade, and >4th Grade. Income level was assessed based on the Portuguese average salary scales and grouped into 3 subcategories: Low (<500€), Average (500€–750€), and High (>750€) income. The residential area was coded as a binary variable (Urban or Rural) following the European Referential Territorial Network [61]. The current spouse status of participants was also coded as binary variables (Yes or No).

#### 2.5.2. Clinical Health Status

The comorbidities were converted into a score using the Charlson comorbidity index [62]. This index measures disease burden as a weighted index based on 19 comorbid conditions. The score, ranging from 0 to 10 and treated as a continuous variable, can be combined with age to form a single index [63]. One point for each additional ten years is added to the initial score, which has been shown to predict 1- and 10-years mortality [62]. Self-reported health status was assessed by the SF-36 questionnaire question: “In general, you would say your health is?”. Self-reported health status was treated as a dichotomous variable, and the participants were classified as having a Negative or Positive health perception [64].

#### 2.5.3. Anthropometrics

Anthropometric and body composition variables were measured in accordance with standard protocols previously described [65], and included the following measures: (i) body mass (Kg), measured by a portable scale with a precision of 0.1 kg (InBody 270, Korea); (ii) stature (cm), determined using a portable stadiometer with a precision of 0.1 cm (Rosscraft, Vancouver, BC, Canada); and (iii) body mass index (BMI), calculated according to the formula BMI = weight/height^2^.

## 3. Data Analysis

Data were analysed with Statistical Package for Social Science Statistics 24 (SPSS Inc., Armonk, NY, USA). The significance level was set at *p* ≤ 0.05. The normality of the continuous variables (age, anthropometric, CCI index, and drug use) was assessed with the Shapiro–Wilk test. Continuous variables were described as mean and standard deviation (SD). Categorical variables data were described by frequency and percentage (%). In the comparison analysis by living place (urban versus rural), Student’s *t*-test and Mann–Whitney (quantitative variables), or Chi-squared (qualitative variables), were computed according to their normality status. Standardised differences between average were reported using Cohen’s *d* effect size values (*ES*) and interpreted as follows: <0.20 (trivial), 0.21 to 0.59 (small), 0.60 to 1.19 (moderate), 1.20 to 1.99 (large), 2.0 to 3.9 (very large), and >4.0 (extremely large) [66]. Spearman’s coefficients were computed to assess correlations among HRQoL overall score PCS and MCS subcomponents, PA summary index, and physical-functional fitness variables of SPBB and HGT tests. The correlations magnitude was interpreted according to the following scores: trivial (*r* < 0.1), small (0.1 < *r* < 0.3), moderate (0.3 < *r* < 0.5), large (0.5 < *r* < 0.7), very large (0.7 < *r* < 0.9), and nearly perfect (*r* > 0.9); positive or negative [66]. The final analysis included regression models using the stepwise method. In model 1 (unadjusted), each component of physical-functional fitness (independent variables) was correlated to HRQoL overall score and physical and mental component summaries (dependent variables), using linear regression analysis. In model 2, a multivariate analysis was performed adjusting the dependent variables of model 1 for sociodemographic, anthropometric, and clinical health co-variates. The collinearity was checked using the variance inflation factor test in all regression models.

## 4. Results

Table 1 shows the sociodemographic, anthropometric, clinical health status, and living place characterization. A total of 259 participants completed the surveys and physical tests and were included in the final analysis. The statistical results indicate that participants living in urban areas were older than their rural counterparts. The total sample consisted mostly of females (*n* = 155, 60%), and the same trend was maintained within each residence group (rural vs. urban).

The majority of the urban (*n* = 70) and rural (*n* = 66) older adults had the 4th-grade schooling level. The number of participants reporting “low income” was higher in urban older individuals (*n* = 64), whereas a higher percentage of urban area residents reported “higher income”. (*n* = 53). A greater percentage of urban residents lived with a partner (*n* = 95), whereas rural residents lived predominantly alone (*n* = 63). No statistical differences were observed regarding the anthropometric variables (weight, height, and BMI) between older adults living in rural and urban areas. In Table 1, the comorbidity index shows significant statistical differences between older people living in rural and urban regions (*p* ≤ 0.05), with participants in urban settings displaying higher values. Consequently, statistical differences were found in medication use. Finally, the majority of both urban (*n* = 132) and rural (*n* = 86) residents claimed to have a positive self-perception of health (<0.001).

Table 2 presents the HRQoL subdimensions, YPAS-p indexes, and functional-physical fitness results of the total sample and subgroups ranked by living place areas. Statistical differences were found for all HRQoL subdimensions, except for general health, mental health, and changes in health status. Likewise, statistical differences were found in the HRQoL overall score, and PCS and MCS subcomponents. These results revealed that, in general, the participants who lived in the rural area had a better HRQoL than urban residents.

Regarding PA levels (and their subdimensions), no statistical differences between the place of residence subgroups were observed in these variables. Statistically significant differences were found in the physical-functional fitness tests, indicating that rural participants had greater SPPB and HGT scores than the urban residents. These results suggest that inhabitants living in rural areas have higher physical-functional fitness levels than urban residents.

Table 3 shows the correlations between physical-functional fitness tests, YPAS-p summary index, and HRQoL (overall score, PCS and MCS subcomponents) by living place. Among older participants living in the urban areas, significant correlations were observed between the YPAS-p summary index and the HRQoL overall score. Positive and moderate correlations were observed between physical-functional tests (SPPB and HGT) and HRQoL overall score, as well as PCS and MCS scores. Concerning rural residents, the YPAS-p summary index presented small correlations with the PCS and HRQoL overall score. Statistically significant moderate and positive correlations emerged among physical-functional tests (SPPB and HGT), and PCS and MCS scores.

Table 4 shows the regression analysis of the association between HRQoL overall score (and its main subdomains), PA levels, and physical-functional fitness of SPPB and HGT in participants living in urban areas. The YPAS-p summary index was significantly associated with HRQoL overall score, explaining 18% of the variance in the HRQoL overall score when covariates are introduced (adjusted model 2) (F (1.126) = 4.353; *p* = 0.040), R^2^ = 0.18). The SPPB total score presented statistical associations with all the three HRQoL variables in both regression models (*p* < 0.001). Moreover, when covariates were introduced in the regression model, the explained variance increased in the association with PCS (19 to 23%), MCS (12 to 14%), and HRQoL overall score (26 to 28%). Considering the three HRQoL variables, the SPPB total score showed a better explanation of the HRQoL overall score in both unadjusted model 1 (F (1.137) = 44.840; *p* < 0.001); R^2^ = 0.26) and adjusted regression model 2 (F (1.126) = 29.949; *p* < 0.001); R^2^ = 0.28).

The HGT also presented a significant correlation with all the three HRQoL variables. When covariates were controlled, the explained variance of HGT increased in the association with PCS (12 to 18%), MCS (0.2 to 14%), and HRQoL (12 to 21%). Additionally, the HGT displayed a better power of variance explanation in the adjusted model 2 of both the PCS subcomponent (F (2.148) = 74.140; *p* < 0.022); R^2^ = 0.18) and HRQoL overall score (F (1.228) 39.976; *p* < 0.001); R^2^ = 0.21).

As depicted in Table 5, in the rural area residents, the YPAS-p summary index was correlated with PCS in the unadjusted (model 1) and HRQoL overall score in both models. When covariates were added to the regression model, the explained variance increased in the association of YPAS-p with the PCS (0.4 to 19%) and HRQoL overall score (0.3 to 28%) subdomains. The better power of variance (28%) emerged from the adjusted model 2 (F (1.185) = 34.820; *p* = 0.000); R^2^ = 0.28). The SPPB score was associated with all the three analysed HRQoL variables in both regression models (*p* < 0.001), except with the MCS in model 2 (*p* = 0.77). In the SPPB scores, the better power of variance emerged from the adjusted model 1, explaining 36% of variance in HRQoL overall score (F (1.158) = 7.875; *p* < 0.001); R^2^ = 0.36). When covariates were introduced, the SPPB-explained variance increased in the association of the PCS (17 to 25%) and the HRQoL (22 to 36%). Finally, the HGT correlated with all HRQoL components, except with the PCS in the adjusted model 2 (*p* = 0.105). Similarly, for urban residents, the explained variance increased when covariates were controlled. The better power of variance was found in adjusted model 2 of HGT, explaining 34% of variance in the HRQoL overall score (F (2.721) = 7.342; *p* = 0.000); R^2^ = 0.34).

## 5. Discussion

This study aimed to assess the perceived HRQoL of older adults living in rural and urban areas, and to examine the influence of PA levels and physical-functional fitness status on their perception. The results indicate three major findings: firstly, the rural residents presented a better HRQoL and physical-functional fitness status than their urban counterparts, corroborating our first hypothesis; the SPPB scores and handgrip strength predicted the HRQoL in both rural and urban older adults residents, partially supporting our second hypothesis; and finally, the predictive power of physical function variables was increased when the models were adjusted for sociodemographic, anthropometric, and clinical health covariates, corroborating the third hypothesis of the study.

### 5.1. Perceived HRQoL of Older Adults Living in Rural and Urban Areas

In the present study, the rural older participants showed better HRQoL than the urban older adults residents. A Portuguese study with a similar population presented contrary results, with older people living in rural areas presenting a lower perceived HRQoL compared to urban residents [67]. The authors mentioned that the low level of education and the lack of systematic (rather than casual) PA may explain some of the reported results. In our study, the higher HRQoL in rural residents may be related to the influence of good clinical health status reported by the participants (high score of self-rated perception, low incidence of comorbidities, and subsequently low intake of medication).

Previous research performed across different countries comparing HRQoL in rural versus urban older residents also reported conflicting results. A study involving Serbian older participants reported a poor HRQoL among urban residents compared to rural residents [43]. Greater poverty, loss of a spouse, life without family members, and lower education were the factors that most contributed to poor HRQoL among the Serbian urban older adults [43]. Similar results were obtained in studies in China (*n* = 9663), Japan (*n* = 880), and Brazil (*n* = 2992) [40,46,68]. In these studies, factors such as lack of opportunities for leisure PA, cultural activities, and attractive outdoor spaces were described as the main contributors to poor HRQoL perception among urban older residents [40,43,46,68].

Some conceptual frameworks have emphasised the influence of sociodemographic, clinical, and psychosocial factors on HRQoL [69]. However, the influence of variables from different domains on HRQoL seems to be more related to lifestyle and life course than perceived HRQoL. In turn, these two dimensions appear to be influenced by health status, socioeconomic status, and education levels in different studies [30,43,46,68]. Therefore, it is expected that the lifestyle adopted by the rural older adults in our study, associated with their autonomy to perform daily tasks (as noticed in the physical-functional fitness test) and environmental factors, may have influenced our results.

### 5.2. Physical Health Status in Rural and Urban Environments

Physical-functional fitness and PA levels are two valid indicators that are highly correlated with physical functioning, and are predictors of autonomy, morbidity, and mortality in older adults [24]. In the present study, the results demonstrated that older individuals from rural areas have higher levels of functional fitness than urban residents. The high physical-functional fitness scores presented by rural residents may be related to the type of daily life activities performed by Portuguese rural residents living in the Inland region of Portugal. Some of these daily tasks require muscle strength and resistance from upper and lower limbs, such as taking care of small farms, and feeding and caring for animals. In accordance with our study, older adults living in Turkish rural areas were more successful at performing physical fitness tests than urban residents [44].

On the other hand, the results of a study conducted in Malaysia, Japan and United States showed that older urban residents had a higher physical-functional fitness status than rural residents [70,71,72]. These differences cannot be explained by the types of tests, since both cited studies present tests very similar to those used in our study. Some of the reasons that may explain the reported differences include the fact that the functional-fitness status of the rural dwellers, especially in developing countries, may be affected by poor access to health care, or the higher levels of physical inactivity in rural areas when compared to metropolitan areas [7,69].

Contrary to what was initially hypothesised, the living environment did not affect the PA levels in our study. Similar results were observed in a study conducted in Turkey [44]. Despite the differences in physical fitness, no significant differences were observed regarding PA levels [44]. On the other hand, an Icelandic study showed that PA levels related to work and daily life tasks were higher in rural residents, whereas PA levels related to leisure were greater in urban dwellers, but no statistical differences were observed between groups [73]. A population-based study conducted in Poland described higher PA levels in urban residents compared to rural residents [33]. In addition, a systematic review of the studies developed in China concluded that the total amount of PA practised by urban residents was relatively low, whereas rural residents presented lower levels of PA, primarily in leisure time [74].

Studies involving the PA assessment in older populations are often controversial [75]. Although there are advantages to self-reported measures (i.e., lower costs and shorter assessment time), there are many challenges regarding the accuracy of the measurements [76]. Some surveys, even after going through validation processes, do not have enough sensitivity to appropriately appraise what is proposed [75], and this fact may explain the results of our study. For example, the study carried out in Poland used a survey that assesses PA related to leisure time activities and motivations, types, exercise, and companions [33]. The Turkey study employed a questionnaire based on the intensity of specific activities, which can be expressed in metabolic equivalents (METs) to estimate energy expenditure in these activities [42]. The researchers of the Iceland study used a brief questionnaire that assessed PA during the past week [71]. In the Canadian Human Activity Pattern Survey, the authors used a more sensitive survey to measure the specific physical outdoor activities carried out in rural areas [32]. In our study, the results revealed that the seniors living in rural areas had higher PA levels compared to urban residents.

Another factor that may have influenced our research results is the low levels of literacy of the participants. Health literacy is considered important in preventing sedentary behaviour and an early deterioration of physical function in older populations [77]. A study recently conducted by our group in Portugal showed that health literacy impacts physical activity levels [78].

### 5.3. Associations between HRQoL, Physical Activity, and Physical-Functional Fitness

Several studies have explored the correlations between HRQoL and indicators of physical health status, providing evidence that physical function parameters, such as PA levels [13,16,17,45] and physical-functional fitness [10,13,16], significantly contribute to older adults’ perceived HRQoL. Considering that muscle strength, gait speed, balance, and lower limb high performance are critical prerequisites for the ability to perform daily living activities independently and prevent falls, it is clear that HRQoL and physical function are strongly linked [10,16,39,46,79].

Our study focused on physical health variables, taking into account the psychobiological paradigm that an active lifestyle and a good functional fitness status influence HRQoL in older populations [64]. As far as we know, this is the first study to look at the correlations between HRQoL, PA levels, and functional-physical fitness status in Portuguese older adults. Our results suggest that functional fitness status is associated with HRQoL, which is in accordance with several of the aforementioned studies. Levels of PA were significantly correlated with the PCS sub-dimension, but did not influence the regression model results. Regardless of these findings, we observed that the inclusion of covariates, such as sociodemographic, clinical health, and anthropometric, influenced the estimated regression model, boosting the explanatory power of physical function variables in the HRQoL and their subdomains.

However, few studies considered the impact of the residential area on the associations between HRQoL and physical health parameters. In a previous study conducted in Poland, a robust correlation between HRQoL and physical-fitness parameters was reported [10]. On the other hand, the authors only analysed the data considering sex differences and did not use covariates in the regression model. In pre-frail and frail older adults, the results of a study conducted in Austria showed that relatively regular PA and high scores in HGT can help older women prevent a decline in HRQoL [17]. Similar to our study, the authors introduced a regression model adjusted for sex and age.

Another study conducted in Brazil [43] reported a moderate correlation between PA levels and HRQoL (physical and mental components). A positive correlation between the PA and HRQoL was found in rural residents. In this study, other aspects, including social interaction, contributed to the perception of a better HRQoL. Recently, a study performed in China revealed that the presence of one or more clinical conditions leading to metabolic diseases was associated with poor HRQoL perception [79]. However, when individuals have good levels of physical activity, the perception of quality of life increases, even with the presence of diseases.

Overall, and in accordance with other studies [43,79], our study presented evidence that physical function is correlated with HRQoL, and that the introduction of some co-variables increased the power of the predicted model. These results are in agreement with previous studies that indicate that related chronic diseases [79], sex [10], age, and education levels [14] may influence HRQoL perception. Moreover, such influence seems to increase with age.

## 6. Limitations and Future Directions

Our work has some limitations. Firstly, this study used a cross-sectional design, which does not allow a causal association. Secondly, since PA and HRQoL were assessed through questionnaires, inaccurate estimation of the PA and HRQoL, and recall bias can be expected. In future research, a more appropriate scale for the assessment of everyday life activities of older people living in rural locations should be adopted. The results of this study cannot be generalised, but indicate that the inclusion of place of residence should be considered as an important factor when analysing the impact of physical health status on HRQoL. Furthermore, longitudinal and randomised controlled trial designs should be performed in the future to garner a deeper understanding of the correlations between physical functioning and HRQoL. The results reported in the current investigation will support the (re)formulation of guidelines that may assist politicians in the development and implementation of strategies to increase physical performance levels, quality of life, and well-being of older adults living in both urban and rural areas. These strategies should include: (i) decentralising and adapting PA programmes according to the needs of the country’s different geographical areas; (ii) creating attractive and innovative PA programmes beyond the “traditional” exercise group fitness class models; (iii) following the biosocial methodological approach in PA intervention programs seeking older adults’ socialisation; (iv) prioritising combined health intervention programmes, aiming at increasing PA, physical-fitness levels, and health literacy for active ageing.

## 7. Conclusions

In this study, the rural residents presented higher HRQoL and functional fitness scores than older adults living in urban areas. The SPPB and handgrip scores influenced the HRQoL overall scores and mental and physical components of rural and urban residents. In contrast, PA levels (measured through the YPAS-p questionnaire) demonstrated a minor influence on the HRQoL overall score. Moreover, the strength of this influence was mediated by covariates such as sociodemographic, anthropometric, and clinical health status variables.

## Figures and Tables

**Table 1:** Characterization of the sociodemographic, anthropometric, and clinical health parameters of the older adults living in urban and rural areas.

	Total Sample (*n* = 259, 100%)	Urban (*n* = 139, 54%)	Rural (*n* = 120, 46%)	*p* Value	Cohen’s *d* ES
*Sociodemographic*					
Chronological age, years (M *±* SD)	74.99 ± 8.08	75.95 ± 7.59	73.87 ± 51	0.020	0.31
Sex, *n* (%) *					
Male	104 (40)	47 (34)	57 (47)	<0.001	--
Female	155 (60)	92 (66)	63 (53)	0.580	
Education, national degree, *n* (%) *					
<4th Grade	50 (20)	9 (7)	41 (34)	<0.001	
=4th Grade	136 (52)	70 (50)	66 (55)		--
>4th Grade	73 (28)	60 (43)	13 (11)		
Income (*n*/%) *					
Low	112 (43)	64 (46)	48 (40)	<0.001	
Medium	43 (17)	24 (17)	19 (15)		--
High	104 (40)	51 (36)	53 (45)		
Living arrangement, *n* (%) *					
Partner	152 (58)	95 (68)	57(48)	<0.001	--
Alone	107 (42)	44 (32)	63 (52)	0.050	
*Anthropometric (M ± SD)*					
Weight (Kg)	74.39 ± 0.71	64.43 ± 11.24	74.38 ± 10.77	0.09	0.17
Height (metres)	1.59 ± 0.64	1.58 ± 0.90	1.60 ± 0.99	0.46	0.09
Body mass index (kg/m^2^)	26.85 ± 3.85	27.11 ± 3.89	26.55 ± 3.79	0.12	0.15
*Clinical Health status (M ± SD)*					
Comorbidities, index	4.06 ± 1.35	4.77 ± 1.33	3.40 ± 1.34	0.012	0.15
Medication use, per day	2.78 ± 1.34	3.80 ± 1.50	2.70 ± 1.16	0.005	0.02
Self-reported health status, *n* (%) *					
Negative	41 (16)	7 (5)	34 (29)	<0.001	--
Positive	218 (84)	132 (95)	86 (71)		

Notes: * Chi-squared test was computed to compare urban vs. rural subgroups; M ± SD = mean and standard deviation; *n* (%) = frequency and percentage; ES = effect size.

**Table 2:** Characterization of health-related quality of life physical activity levels and physical-functional fitness status according to the living place of the participants.

	Total Sample (*n* = 259, 100%)	Urban (*n* = 139, 54%)	Rural (*n* = 120, 46%)	*p* Value	Cohen’s *d* ES
*Health-related quality of life by SF-36 (M ± SD)*					
Physical functioning	68.21 ± 28.21	64.10 ± 28.27	72.97 ± 27.50	0.000	0.32
Physical health	65.51 ± 28.24	62.27 ± 29.45	69.27 ± 26.39	0.022	0.32
Bodily pain	64.07 ± 30.18	61.12 ± 29.92	67.49 ± 30.25	0.050	0.25
General health	56.36 ± 18.88	55.71 ± 19.40	57.10 ± 18.32	0.281	0.08
Vitality	63.70 ± 18.36	62.05 ± 18.30	65.62 ± 18.33	0.050	0.20
Social functioning	75.33 ± 22.53	72.03 ± 22.67	79.16 ± 20.74	0.017	0.32
Emotional health	70.20 ± 27.94	67.50 ± 29.23	73.33 ± 16.14	0.050	0.21
Mental health	65.40 ± 20.40	64.06 ± 21.72	66.95 ± 19.21	0.133	0.14
Changes in health status	52.12 ± 19.81	52.87 ± 21.71	51.25 ±17.40	0.252	0.08
Physical component summary	49.01 ± 10.39	48.86 ± 10.20	51.31 ± 10.50	0.031	0.24
Mental component summary	50.09 ± 9.57	49.12 ± 10.14	51.01 ± 8.79	0.050	0.20
SF-36 overall score	48.07 ± 7.70	48.99 ± 7.78	51.16 ± 7.48	0.012	0.28
*Levels of Physical Activity by YPAS-p (M ± SD)*					
Vigorous index (units/month)	8.94 ± 14.85	8.69 ± 7.33	9.24 ± 5.48	0.381	0.03
Leisure walk index (units/month)	13.65 ± 10.49	13.72 ± 9.62	13.56 ± 8.38	0.462	0.01
Moving index (hours/day)	9.00 ± 3.43	8.91 ± 3.34	9.10 ± 3.54	0.332	0.05
Standing index (hours/day)	6.14 ± 2.16	6.08 ± 2.02	6.22 ± 2.33	0.314	0.06
Sitting index (hours/day)	2.19 ± 0.88	2.25 ± 0.90	2.13 ± 0.84	0.143	0.13
Summary index (total units)	36.26 ± 25.83	35.01 ± 27.59	37.31 ± 14.65	0.201	0.10
*Functional-physical fitness (M ± SD)*					
SPPB overall score	9.86 ± 1.93	9.69 ± 1.95	11.07 ± 1.90	0.050	0.20
Handgrip strength test (kg)	26.08 ± 8.93	25.03 ± 8.11	27.31 ± 9.68	0.019	0.26

Notes: M ± SD = mean and standard deviation; ES = effect size; SPPB = short physical performance battery; YAPS-p = Portuguese version of Yale physical activity survey; SF-36 = Short Form Health Survey.

**Table 3:** Spearman’s correlations between physical-functional tests and health-related quality of life according to the living place.

	PCS	MCS	HRQoL Overall Score
*Urban (n = 139)*			
YPAS-p summary index	0.15	0.15	0.19 *
SPBB total score	0.44 **	0.35 *	0.52 **
Handgrip strength test	0.36 **	0.28 *	0.36 **
*Rural (n = 120)*			
YPAS-p index	0.22 *	0.07	0.20 *
SPBB total score	0.30 **	0.27 **	0.31 **
Handgrip test	0.43 **	0.38 **	0.52 **

Notes: * *p* ≤ 0.05; ** *p* ≤ 0.01; YPAS-p = Portuguese version of Yale physical activity scale; SPPB = short physical fitness test battery; PCS = physical component summary; MCS = mental component summary; HrQoL = health-related quality of life.

**Table 4:** Regression models for physical-functional fitness tests (SPPB and HGT) and physical activity levels (YPAS-p indexes) predicting health-related quality of life of older participants living in urban areas (*n* = 139).

	PCS			MCS			HRQoL Overall Score		
	R^2^	β	*p*	R^2^	β	*p*	R^2^	β	*p*
*YPAS-p summary index*									
Model 1	0.02	0.94	0.271	0.02	0.15	0.830	0.13	0.16	0.633
Model 2	0.15	0.33	0.282	0.23	0.63	0.491	0.18	0.04	0.050
*SPPB total score*									
Model 1	0.19	2.31	<0.001	0.12	0.33	<0.001	0.26	0.51	<0.001
Model 2	0.23	1.76	<0.001	0.14	1.97	<0.001	0.28	1.87	<0.001
*Handgrip strength test*									
Model 1	0.12	0.45	<0.001	0.02	0.18	0.036	0.12	0.36	<0.001
Model 2	0.18	0.34	0.022	0.14	0.60	0.001	0.21	0.45	<0.001

Notes: Model 1 included one dependent and one independent variable; Model 2 included the adjustment for sociodemographic (sex, education, income, and living arrangement) and clinical health status (comorbidities, medication use, and self-rated health); YPAS-p = Portuguese version of Yale Physical Activity Scale; SPPB = short physical fitness test battery; PCS = physical component summary; MCS = mental component summary; HrQoL = health-related quality of life.

**Table 5:** Regression models for physical-functional fitness tests (SPPB and HGT) and physical activity levels (YPAS-p indexes) predicting health-related quality of life of older participants living in rural areas (*n* = 120).

	PCS			MCS			HRQoL		
	R^2^	β	*p*	R^2^	β	*p*	R^2^	β	*p*
*YPAS-p summary index*									
Model 1	0.04	0.22	0.021	0.00	0.74	0.421	0.03	0.19	0.032
Model 2	0.19	0.54	0.163	0.15	−0.10	0.764	0.28	0.05	0.000
*SPPB overall score*									
Model 1	0.17	2.32	<0.001	0.10	0.31	<0.001	0.22	0.48	<0.001
Model 2	0.25	1.65	<0.001	0.19	0.09	0.77	0.36	1.31	<0.001
*Handgrip strength test*									
Model 1	0.17	0.46	<0.001	0.14	0.37	<0.001	0.26	0.51	<0.001
Model 2	0.22	0.38	0.002	0.17	0.20	0.105	0.34	0.30	0.000

Notes: Model 1 included one dependent and one independent variable; Model 2 included the adjustment for sociodemographic (sex, education, income, and living arrangement) and clinical health status (comorbidities, medication use, and self-rated health); YPAS-p = Portuguese version of Yale physical activity scale; SPPB = short physical fitness test battery; PCS = physical component summary; MCS = mental component summary; HrQoL = health-related quality of life.

## Data Availability

The data presented in this study are available on request from the corresponding author. The data are not publicly available due to privacy restrictions.
